# Comprehensive Benchmark Dataset for Pathological Lymph Node Metastasis in Breast Cancer Sections

**DOI:** 10.1038/s41597-025-05586-5

**Published:** 2025-08-07

**Authors:** Xitong Ling, Yuanyuan Lei, Jiawen Li, Junru Cheng, Wenting Huang, Tian Guan, Jian Guan, Yonghong He

**Affiliations:** 1 Shenzhen International Graduate School, Tsinghua University, Shenzhen, 518071 China; 2 National Cancer Center/National Clinical Research Center for Cancer/Cancer Hospital & Shenzhen Hospital, Chinese Academy of Medical Sciences and Peking Union Medical College, Shenzhen, 518116 China; 3 Research Institute of Tsinghua, Guangzhou, 508157 China

**Keywords:** Breast cancer, Medical imaging

## Abstract

Advances in optical microscopy scanning have significantly contributed to computational pathology (CPath) by converting traditional histopathological slides into whole slide images (WSIs). This development enables comprehensive digital reviews by pathologists and accelerates AI-driven diagnostic support for WSI analysis. Recent advances in foundational pathology models have increased the need for benchmarking tasks. The Camelyon series is one of the most widely used open-source datasets in computational pathology. However, the quality, accessibility, and clinical relevance of the labels have not been comprehensively evaluated.In this study, we reprocessed 1,399 WSIs and labels from the Camelyon-16 and Camelyon-17 datasets, removing low-quality slides, correcting erroneous labels, and providing expert pixel annotations for tumor regions in the previously unreleased test set. Based on the sizes of re-annotated tumor regions, we upgraded the binary cancer screening task to a four-class task: negative, micro-metastasis, macro-metastasis, and Isolated Tumor Cells (ITC). We reevaluated pre-trained pathology feature extractors and multiple instance learning (MIL) methods using the cleaned dataset, providing a benchmark that advances AI development in histopathology.

## Background & Summary

The efficient utilization of digital pathology and computational resources has led to the rapid rise of AI-based computational pathology^1,2^. In recent years, general foundational models for pathology, pre-trained on large-scale data, have garnered significant attention^3–7^. These models have demonstrated strong feature extraction capabilities for pathological images, as evidenced by evaluations across a series of whole-slide image-level downstream tasks^8–10^. For example, CTranspath^6^ uses a Semantically-Relevant Contrastive Learning (SRCL) framework to pre-train a CNN-Transformer hybrid feature extractor on 150 million patches, with its effectiveness validated across five downstream tasks. UNI^4^ employed the self-supervised DINO-v2^11^ method to train a robust general pathology visual encoder on one billion patches from approximately 100,000 whole slide images (WSIs). Gigapath^5^ utilized 1.3 billion patches to pre-train a visual encoder based on VIT-Gaint^12^ architecture and adopted LongNet^13^ to scale itself to a slide-level foundation model for slide-level representation learning. Virchow^14^ is a pathology foundation model based on the ViT-Huge architecture, trained using the DINOv2 approach on a dataset constructed from 1,488,550 whole slide images (WSIs), enabling clinical-grade diagnosis and rare disease identification. Pathorchestra^15^ trained a VIT-Large encoder on 300,000 WSIs and conducted extensive evaluation across 112 downstream tasks, achieving over 95% accuracy on 47 of them. These pathology-pre-trained models have demonstrated superior performance in downstream tasks including tumor classification, survival analysis, and lesion segmentation. PLIP^3^, pre-trained on approximately 200,000 pathology image-text pairs collected from medical Twitter, developed a multimodal pathology foundational model using contrastive learning^16^, capable of both image and text comprehension. CONCH^7^ employs CoCa^17^ for self-supervised pre-training on 1.17 million image-caption pairs and has been extensively evaluated across 14 downstream benchmarks, demonstrating its outstanding performance. In addition to patch-level encoders, some studies have focused on developing pretrained slide-level encoders, which are built upon patch encoders. For example, CHIEF^18^ constructs a slide encoder with an ABMIL^19^ architecture through vision-language joint training based on CTranspath^6^, using 60,530 WSIs. Prism^20^ is a Transformer-based slide encoder trained on 587,196 WSIs, built upon patch embeddings from Virchow. Titan^21^ is a slide-level encoder trained via slide-level vision-language contrastive learning, based on CONCH-V1.5^21^, an upgraded version of the CONCH model. Slide-level encoders eliminate the need to retrain aggregators by directly generating WSI-level representations through inference, enabling downstream slide-level tasks such as classification, survival analysis, and report generation.

Acquiring finely annotated large-scale pathology image datasets remains challenging due to the extremely high resolution of pathology images and the specialized expertise required for annotations. Nonetheless, the continued development of foundational models and downstream tasks in computational pathology makes high-quality pathology image datasets increasingly essential.

**Fig. 1:**
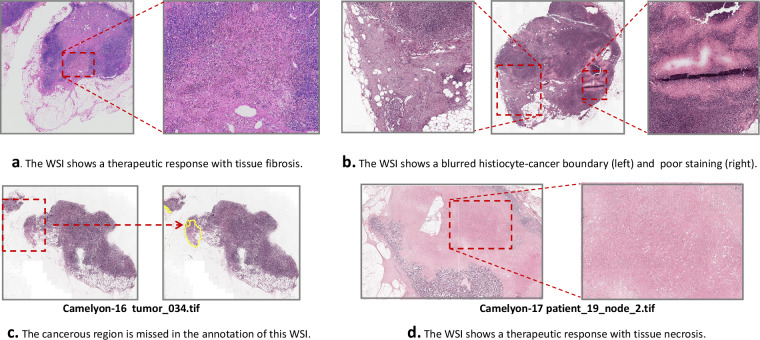
Examples of issues in the Camelyon-16 and Camelyon-17 datasets. **(a)** The WSI shows a therapeutic response characterized by tissue fibrosis. **(b)** The WSI exhibits a blurred histiocyte-cancer boundary (left) and poor staining quality (right). **(c)** The cancerous region is missed in the annotation. **(d)** The WSI shows a therapeutic response with tissue necrosis.

The Camelyon series^22,23^ (http://gigadb.org/dataset/100439), a publicly available pathology dataset focused on detecting breast cancer lymph node metastasis, is widely used for evaluating multiple instance Learning (MIL) methods. However, as shown in Fig. 1, some images in the Camelyon series are of poor quality, exhibit treatment-related artifacts, and contain errors in slide-level labeling. The Camelyon-16^22^ dataset includes only tumor and negative labels, making it incompatible with Camelyon-17^23^ labels. Many pixel-level annotations are inaccurate, and some slides lack pixel-level annotations entirely. These issues hinder the accurate evaluation of deep learning methods in downstream pathology tasks.

In this paper, we filtered out and removed slides from the Camelyon dataset that were blurred, poorly stained, exhibited treatment-related artifacts, or were ambiguous in terms of positivity. Furthermore, we expanded the binary classification labels in Camelyon-16^22^ to a four-class system to facilitate the merging of the Camelyon-16 and Camelyon-17^23^ datasets. Finally, we corrected the pixel-level annotations in the Camelyon dataset and added pixel-level annotations to positive slides that previously lacked them. Using the corrected dataset, we reevaluated 12 main MIL methods, including ABMIL^19^, TransMIL^24^ and CLAM^25^, etc. in two pre-trained natural image feature encoders, ResNet-50^26^ and VIT-S^12^, as well as ten pathology-specific pre-trained feature encoders, PILP^3^, CONCH^7^, UNI^4^, Gigapath^5^, CONCH-V1.5^21^, TITAN^21^, Virchow^14^, Prism^20^, Ctranspath^6^ and Chief^18^.

## Technical Validation

### Dataset Overview

**Fig. 2:**
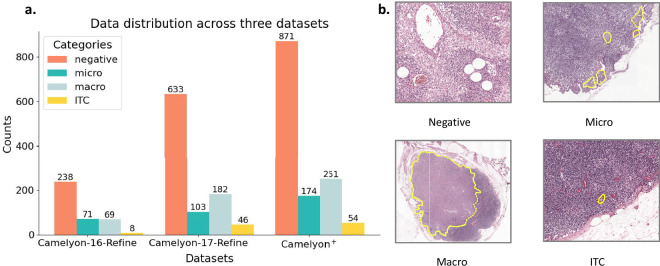
Data characteristics and metastasis categories in Camelyon datasets. **(a)** Distribution of WSIs across different metastasis categories (Negative, Micro, Macro, and ITC) in three datasets: Camelyon-16-Refine^22^, Camelyon-17-Refine^23^, and Camelyon^+^^27^. **(b)** Representative histopathological examples for each category: Negative, Micro, Macro, and ITC.

The official Camelyon-16^22^ dataset contains 399 WSIs, split into 270 for training and 129 for testing. The training set includes 111 tumor slides and 259 negative slides, while the test set includes 49 tumor slides and 80 negative slides. The official Camelyon-17^23^ dataset consists of 1000 WSIs, evenly divided into 500 for training and 500 for testing. The training set consists of 318 negative slides, 59 micro-metastasis slides, 87 macro-metastasis slides, and 36 Isolated Tumor Cells (ITC) slides. The test set labels are not publicly available. After data cleaning by professional pathologists, the Camelyon-16 dataset consists of 386 WSIs: 238 negative, 71 micro-metastasis, 69 macro-metastasis, and 8 ITC WSIs. The Camelyon-17 dataset consists of 964 WSIs: 633 negative, 103 micro-metastasis, 182 macro-metastasis, and 46 ITC WSIs. We combined the updated Camelyon-16 and Camelyon-17 datasets to form the Camelyon^+^ dataset. Figure 2 shows the dataset overvire. It consists of 1,350 WSIs: 871 negative, 174 micro-metastasis, 251 macro-metastasis, and 54 ITC WSIs.

### Exclusion Criteria

We excluded certain WSIs based on the following criteria: focal blurriness, poor staining quality, difficulty distinguishing positive foci, and the presence of treatment-related artifacts. Of the 49 slides we remove, 26 show therapeutic response, 3 have staining issues, 12 exhibit focal blurring, 4 are of poor quality, and 4 contain suspicious cancerous regions. The verification of WSI labels and the annotation work were performed in the ASAP pathology annotation software (https://computationalpathologygroup.github.io/ASAP) by a mid-level pathologist, in accordance with the 8th edition of the American Joint Committee on Cancer (AJCC), and consistency checks were conducted by a senior pathologist. The presence of treatment response may interfere with model construction. In pathology, tumor treatment response refers to the histological changes in tumors following treatments such as surgery, chemotherapy, radiotherapy, targeted therapy, or immunotherapy, and the corresponding reaction to these treatments. Pathological analysis can assess histological indicators such as tumor cell necrosis, proliferation, and apoptosis, thereby evaluating treatment efficacy. Two typical treatment responses are tissue necrosis and fibrosis. Necrosis refers to areas of dead tissue formed after tumor cells die following treatment. Fibrosis refers to the scar tissue formed as a result of the self-repair of the tumor after damage. Necrotic and fibrotic areas can affect the representation of the characteristics of tumor regions in computational pathology, thereby affecting the performance of downstream tasks.

## Data Records

The Camelyon^+^^27^ dataset is available via ScienceDB: (10.57760/sciencedb.16442). The original WSI data can be downloaded from the official dataset repository (http://gigadb.org/dataset/100439), so it has not been uploaded to the database. The Camelyon^+^ dataset is structured into several directories, each serving a specific function in supporting downstream computational pathology tasks. The directory structure includes the following main components: slide-labels/, name-convert/, pixel-annotations/, feature-files/, and h5py-files/.

- slide-labels/: This directory stores slide-level classification annotations in Excel format. We provide two XLSX files: camelyon+(2-classes).xlsx and camelyon+(4-classes).xlsx, which correspond to binary classification (negative vs. tumor) and four-class classification (negative, micro, macro, ITC), respectively. Each file contains two columns: slide (the WSI ID) and label (the assigned class). These labels are derived from corrected and unified versions of the Camelyon-16 and Camelyon-17 datasets, supporting various supervised learning scenarios.
- name-convert/: To eliminate annotation bias, all original WSI file names from the Camelyon-16 training set that contain diagnostic hints such as “tumor” or “normal” have been renamed. The name-convert.xlsx file in this directory provides a mapping between the original and new file names through two columns: Origin Name and New Name. This enables accurate cross-referencing during label alignment or post-hoc analysis.
- pixel-annotations/: For positive WSIs, pixel-level tumor region annotations are provided in XML format. Polygonal coordinates of positive regions are stored in the XML files and can be visualized on whole-slide images using ASAP. These files include detailed boundary information and can be used for tasks such as semantic segmentation or weakly supervised learning.
- feature-files/: To facilitate fair and reproducible benchmarking across various visual encoders, this directory contains patch-level features extracted at 20 × magnification using a diverse set of backbone models, including ResNet-50^26^, VIT-S^12^, PLIP^3^, CONCH^7^, CONCH-V1.5^21^, Ctranspath^6^, UNI^4^, GigaPath^5^, Virchow^14^, Chief^18^, Prism^20^, and Titan^21^. All features are stored in .pt format, which is natively compatible with the PyTorch library and supports efficient loading during training and inference.
- h5py-files/: This optional directory offers an alternative representation of extracted features in .h5 format, enabling high-speed access and batch-wise loading for large-scale training workflows.

This modular organization of Camelyon^+^^27^ supports a broad spectrum of tasks, including classification, segmentation, and representation learning, and provides a standardized testbed for developing and evaluating pathology foundation models.

## Methods

### Methodology

The objective of our designed benchmark is to utilize slide-level labels to predict metastasis types. The commonly used approach is to adopt a deep learning strategy based on MIL, which has been recognized in recent studies for its strong capability to represent slide-level features^28–32^. MIL is a weakly supervised approach where a single WSI is treated as a bag, and each patch within the WSI is considered an instance. If any instance is cancerous, the entire WSI is labeled as cancerous, while a WSI is classified as normal only if all instances are normal.

With the advancement of deep neural networks, embedding-based MIL has become the dominant approach for WSI analysis. In embedding-based MIL, a pre-trained feature extractor first extracts features from the WSI, followed by an aggregator that pools the features for downstream classification tasks. Mean-MIL and Max-MIL aggregate features using mean pooling and max pooling, respectively, though the pooling mechanism inevitably results in information loss. ABMIL^19^ introduces the attention mechanism into MIL, dynamically assigning weights to each instance based on attention scores. CLAM^25^ further enhances this by incorporating instance-level clustering mechanisms to introduce domain knowledge, providing additional supervision alongside attention-based weight assignments. TransMIL^24^ leverages self-attention within the MIL aggregator to capture relationships between different instances, thereby improving global modeling capabilities. AMD-MIL^33^ introduces an agent mechanism into the MIL aggregator and employs threshold filtering for feature selection, improving MIL performance. DSMIL^34^ models instance relationships directly using a dual-stream architecture and a trainable distance measurement module. DTFD^35^ addresses the issue of limited WSIs by creating pseudo-bags. WiKG^36^ treats WSIs as knowledge graphs, dynamically constructing neighboring nodes and directed edges based on relationships between instances, and then updates the head node using knowledge-aware attention. FR-MIL^37^ introduces a distribution re-calibration approach that adjusts the feature distribution of a WSI bag (instances) based on the statistics of the max-instance (key) feature.

### Data Preprocessing

For all datasets, we crop non-overlapping 256 × 256 patches at 20 × magnification. We then use twelve feature extractors ResNet-50^26^, VIT-S^12^, PILP^3^, CONCH^7^, UNI^4^, Gigapath^5^, CONCH-V1.5^21^, TITAN^21^, Virchow^14^, Prism^20^, Ctranspath^6^ and Chief^18^ to extract features from the WSIs. Subsequently, we conducted two sets of experiments. The first set is a comparative experiment on the Camelyon-17^23^ dataset before and after label correction. The Camelyon-17-Origin dataset follows the official split, with 500 WSIs for training and 500 WSIs for testing. The Camelyon-17-Refine dataset also maintains the official split but excludes slides that fall under exclusion criteria. The Camelyon-17-Refine training set contains 492 slides, while the test set includes 472 slides. This comparative experiment evaluates the impact of dataset quality on MIL models. Since the original version of Camelyon-16^22^ does not have four-class labels, we do not perform similar experiments on it. The next set is the benchmark experiments on Camelyon^+^^27^. We evaluate using five-fold cross-validation, with each fold employing stratified sampling to maintain a fixed proportion of different classes. Since each patient has multiple slides in the Camelyon-17 dataset, in order to prevent data leakage, we ensure that slides of the same label of the same patient do not appear in the training set and the validation set at the same time.

### Camelyon-17 Comparative Experiment

**Fig. 3:**
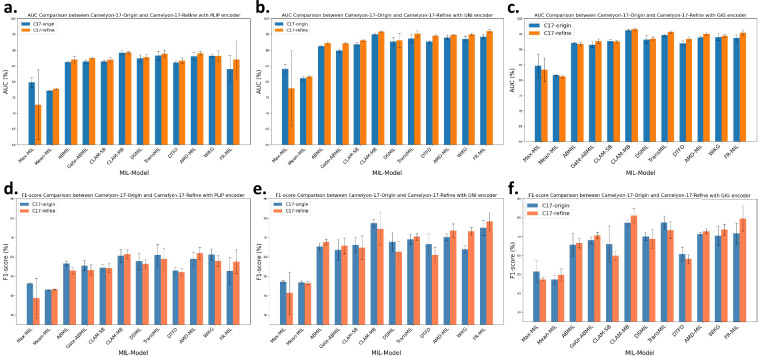
Performance comparison between Camelyon-17-Origin^23^ and Camelyon-17-Refine across different MIL models and feature encoders. **(a–c)** AUC comparison using three feature encoders: **(a)** PLIP^3^, **(b)** UNI^4^, and **(c)** Gigapath^5^. **(d–f)** F1-score comparison using three feature encoders: **(d)** PLIP, **(e)** UNI, and **(f)** Gigapath.

**Fig. 4:**
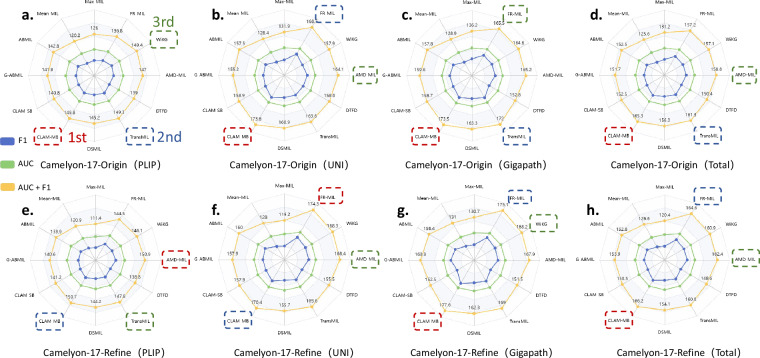
Radar chart analysis of MIL models across different encoders and dataset versions. Each subplot shows the performance of multiple MIL models on the Camelyon-17-Origin^23^ and Camelyon-17-Refine datasets using three feature encoders: **(a,e)** PLIP^3^, **(b,f)** UNI^4^, and **(c,g)** Gigapath^5^. The outermost yellow line represents the average of AUC and F1-score, green represents AUC, and blue represents F1-score. **(d,h)** summarize the total ranking across all encoders for each dataset. Top-3 ranked models are highlighted with dashed boxes.

In the comparative experiments before and after correction on the Camelyon-17^23^ dataset, we primarily evaluated three pathology pre-trained feature extractors: PLIP^3^, UNI^4^, and Gigapath^5^. The learning rate was set to 2e-4, using the Adam optimizer with a weight decay of 1e-5. We repeated the experiments with random seeds of 2023, 2024, and 2025, and reported the mean and standard deviation of the evaluation metrics as shown in Table 1 and Table 2. All experiments were conducted on a workstation equipped with 4 NVIDIA RTX 3090 GPUs. Due to the significant class imbalance in the Camelyon-17 four-class dataset, our analysis concentrated on two key evaluation metrics: AUC and F1-score. Figure 3 presents a visualization of these metrics for a single MIL model across different feature extractors, using bar charts for both the Camelyon-17-Origin and Camelyon-17-Refine datasets. This visualization effectively illustrates how these metrics vary as the dataset undergoes refinement. Our results indicate that both AUC and F1-score exhibited notable changes following the dataset’s adjustment. Figure 4 further visualizes the F1-score, AUC, and their combined values, highlighting the top three models to assess the impact of dataset refinement on the performance ranking of the models. While the overall model rankings shifted to some extent after the dataset refinement, the CLAM-MB^25^ model consistently maintained its top-ranked position, indicating its robustness. In summary, dataset refinement enhanced the accuracy of model evaluation metrics and improved the fairness of model rankings, establishing a more solid foundation for future research.

**Table 1:** Performance metrics of different methods on the Camelyon-17-Origin dataset.

Methods	Acc (%)	AUC (%)	F1 (%)	Recall (%)	Precision (%)	Kappa
PLIP^3^ (WSIs pre-trained)						
Max-MIL	83.1 ± 0.90	79.8 ± 1.58	46.2 ± 0.34	45.4 ± 0.83	46.6 ± 2.35	0.69 ± 0.00
Mean-MIL	78.7 ± 0.64	77.1 ± 0.17	43.1 ± 0.25	44.5 ± 0.43	49.3 ± 1.58	0.56 ± 0.01
ABMIL^19^	84.4 ± 0.72	86.2 ± 0.24	56.6 ± 1.17	54.4 ± 0.31	53.5 ± 0.45	0.77 ± 0.01
Gate-ABMIL^19^	83.9 ± 1.30	86.4 ± 0.50	55.5 ± 2.66	53.8 ± 1.78	53.3 ± 0.69	0.76 ± 0.02
CLAM-SB^25^	84.9 ± 0.50	86.4 ± 0.32	54.4 ± 1.63	53.2 ± 1.05	52.5 ± 0.55	0.76 ± 0.01
CLAM-MB^25^	86.1 ± 0.42	**89.2** ± **0.62**	60.6 ± 3.30	58.4 ± 2.58	59.4 ± 5.25	**0.79** ± **0.02**
DSMIL^34^	86.1 ± 0.61	87.4 ± 1.14	57.8 ± 4.20	57.9 ± 3.57	**61.2** ± **6.58**	0.76 ± 0.02
TransMIL^24^	85.4 ± 1.11	88.3 ± 1.42	61.0 ± 5.51	58.3 ± 4.43	56.7 ± 4.24	0.74 ± 0.06
DTFD^35^	85.5 ± 0.50	86.1 ± 0.55	52.9 ± 1.79	52.1 ± 2.14	52.6 ± 1.01	0.76 ± 0.01
AMD-MIL^33^	83.7 ± 3.18	88.1 ± 1.31	58.9 ± 3.40	56.4 ± 4.36	55.1 ± 4.74	0.73 ± 0.02
WiKG^36^	**86.3** ± **1.22**	88.2 ± 0.41	**61.2** ± **2.91**	**59.1** ± **3.13**	58.4 ± 3.04	0.78 ± 0.01
FR-MIL^37^	82.7 ± 3.64	84.0 ± 4.32	52.8 ± 6.72	52.0 ± 5.30	52.1 ± 4.52	0.71 ± 0.07
UNI^4^ (WSIs pre-trained)						
Max-MIL	85.3 ± 0.58	84.1 ± 1.40	47.2 ± 0.59	44.8 ± 0.75	45.0 ± 4.80	0.72 ± 0.03
Mean-MIL	76.1 ± 2.66	81.1 ± 0.55	46.8 ± 0.74	47.0 ± 1.25	48.4 ± 2.37	0.57 ± 0.03
ABMIL^19^	82.4 ± 0.35	91.3 ± 0.35	65.4 ± 1.86	61.2 ± 1.35	59.7 ± 1.63	0.73 ± 0.02
Gate-ABMIL^19^	80.9 ± 1.40	89.9 ± 0.44	63.6 ± 5.47	58.8 ± 4.05	56.7 ± 3.37	0.71 ± 0.02
CLAM-SB^25^	81.3 ± 1.86	91.9 ± 0.53	66.2 ± 4.04	59.9 ± 2.85	58.5 ± 1.61	0.70 ± 0.04
CLAM-MB^25^	85.1 ± 0.31	**95.0** ± **0.41**	**77.4** ± **2.02**	**68.1** ± **1.20**	**64.9** ± **0.55**	0.70 ± 0.02
DSMIL^34^	83.2 ± 0.53	92.7 ± 1.27	67.7 ± 4.95	61.8 ± 3.23	60.2 ± 2.49	0.66 ± 0.05
TransMIL^24^	**86.0** ± **0.35**	93.7 ± 1.24	69.0 ± 2.47	64.4 ± 2.31	64.4 ± 1.92	**0.77** ± **0.02**
DTFD^35^	84.4 ± 0.60	92.7 ± 0.37	66.6 ± 5.33	61.7 ± 5.15	59.2 ± 4.56	0.77 ± 0.04
AMD-MIL^33^	82.5 ± 1.62	94.0 ± 0.70	70.2 ± 1.79	63.0 ± 1.60	61.1 ± 1.04	0.63 ± 0.06
WiKG^36^	84.6 ± 3.20	93.6 ± 0.87	63.9 ± 1.87	60.0 ± 1.72	58.4 ± 1.25	0.72 ± 0.08
FR-MIL^37^	84.1 ± 0.70	94.3 ± 0.66	75.0 ± 3.91	66.2 ± 2.13	63.5 ± 1.28	0.71 ± 0.01
Gigapath^5^ (WSIs pre-trained)						
Max-MIL	83.5 ± 1.81	84.7 ± 3.79	51.5 ± 5.76	49.5 ± 5.31	48.2 ± 4.85	0.74 ± 0.04
Mean-MIL	78.3 ± 1.36	81.6 ± 0.17	47.3 ± 2.31	48.7 ± 1.69	53.2 ± 3.11	0.53 ± 0.05
ABMIL^19^	79.2 ± 2.43	92.1 ± 0.27	65.7 ± 6.11	58.1 ± 5.31	58.6 ± 0.99	0.68 ± 0.03
Gate-ABMIL^19^	80.7 ± 1.14	91.6 ± 0.99	68.0 ± 1.85	61.9 ± 1.88	60.0 ± 1.55	0.72 ± 0.02
CLAM-SB^25^	80.3 ± 4.02	92.7 ± 0.59	66.0 ± 9.69	60.1 ± 8.21	61.4 ± 4.20	**0.75** ± **0.01**
CLAM-MB^25^	85.5 ± 0.61	**96.2** ± **0.30**	77.3 ± 1.45	68.3 ± 0.94	**65.2** ± **0.73**	0.70 ± 0.02
DSMIL^34^	**86.2** ± **1.71**	93.2 ± 1.30	70.1 ± 2.06	65.6 ± 0.86	63.6 ± 1.00	0.71 ± 0.01
TransMIL^24^	84.7 ± 2.21	94.6 ± 0.36	**77.4** ± **3.45**	**68.3** ± **3.26**	64.6 ± 3.06	0.73 ± 0.04
DTFD^35^	81.5 ± 1.22	92.0 ± 0.80	60.8 ± 3.69	55.6 ± 3.66	54.2 ± 2.66	0.75 ± 0.07
AMD-MIL^33^	83.5 ± 1.03	93.9 ± 0.36	71.3 ± 0.79	64.7 ± 0.38	62.9 ± 1.31	0.74 ± 0.01
WiKG^36^	83.4 ± 0.72	94.0 ± 1.13	70.6 ± 5.09	64.5 ± 2.23	63.0 ± 1.46	0.74 ± 0.05
FR-MIL^37^	83.9 ± 0.81	93.7 ± 1.02	71.8 ± 5.51	64.8 ± 3.34	62.3 ± 2.78	0.70 ± 0.04

**Table 2:** Performance metrics of different methods on the Camelyon-17-Refine dataset.

Methods	Acc (%)	AUC (%)	F1 (%)	Recall (%)	Precision (%)	Kappa
PLIP^3^ (WSIs pre-trained)						
Max-MIL	77.8 ± 9.55	72.7 ± 11.09	38.7 ± 10.28	37.4 ± 10.63	41.0 ± 3.25	0.49 ± 0.32
Mean-MIL	79.8 ± 0.24	77.7 ± 0.25	43.2 ± 0.18	44.8 ± 0.22	49.2 ± 0.57	0.59 ± 0.00
ABMIL^19^	81.8 ± 1.39	87.0 ± 1.18	52.9 ± 1.63	51.0 ± 1.48	51.5 ± 0.42	0.75 ± 0.02
Gate-ABMIL^19^	81.9 ± 2.04	87.5 ± 0.13	53.1 ± 2.83	51.3 ± 2.61	51.7 ± 1.01	0.75 ± 0.02
CLAM-SB^25^	82.8 ± 1.73	87.0 ± 0.76	54.2 ± 2.32	52.3 ± 1.95	52.0 ± 0.88	0.76 ± 0.02
CLAM-MB^25^	**87.2** ± **0.80**	**89.3** ± **0.41**	61.4 ± 2.12	**59.6** ± **2.69**	**61.2** ± **7.85**	**0.82** ± **0.01**
DSMIL^34^	86.2 ± 1.32	87.8 ± 0.86	56.4 ± 2.37	56.2 ± 1.95	56.5 ± 1.79	0.76 ± 0.02
TransMIL^24^	83.8 ± 1.00	88.9 ± 1.15	58.9 ± 5.70	56.9 ± 4.14	56.7 ± 2.78	0.71 ± 0.02
DTFD^35^	84.3 ± 1.81	86.7 ± 0.95	52.1 ± 1.79	51.2 ± 1.12	51.3 ± 0.19	0.77 ± 0.01
AMD-MIL^33^	86.4 ± 0.97	89.0 ± 0.67	**61.9** ± **3.01**	59.6 ± 3.07	58.4 ± 2.66	0.78 ± 0.03
WiKG^36^	87.1 ± 1.20	88.2 ± 1.64	57.9 ± 2.76	56.6 ± 2.13	55.8 ± 1.86	0.80 ± 0.03
FR-MIL^37^	80.6 ± 1.59	87.0 ± 5.90	57.5 ± 6.19	55.3 ± 4.36	54.8 ± 3.49	0.63 ± 0.02
UNI^4^ (WSIs pre-trained)						
Max-MIL	79.6 ± 10.07	77.9 ± 11.95	41.3 ± 10.87	39.1 ± 9.87	41.1 ± 2.10	0.55 ± 0.35
Mean-MIL	76.5 ± 2.02	81.6 ± 0.26	46.4 ± 0.70	46.8 ± 1.09	49.4 ± 0.83	0.49 ± 0.06
ABMIL^19^	82.1 ± 1.60	92.3 ± 0.64	67.7 ± 1.51	61.9 ± 1.47	59.6 ± 1.29	0.72 ± 0.02
Gate-ABMIL^19^	81.0 ± 0.86	92.2 ± 0.28	65.7 ± 4.13	59.7 ± 3.14	57.9 ± 2.48	0.68 ± 0.05
CLAM-SB^25^	81.3 ± 1.07	93.1 ± 0.22	64.8 ± 6.08	58.5 ± 4.25	56.5 ± 3.85	0.73 ± 0.07
CLAM-MB^25^	85.0 ± 0.68	**95.9** ± **0.21**	74.5 ± 8.42	65.6 ± 3.72	63.4 ± 3.06	0.70 ± 0.08
DSMIL^34^	85.9 ± 3.17	93.1 ± 2.26	62.6 ± 5.28	59.9 ± 3.16	59.0 ± 1.35	0.75 ± 0.05
TransMIL^24^	**88.5** ± **0.44**	95.2 ± 0.93	70.4 ± 1.88	65.7 ± 1.97	66.1 ± 6.04	0.78 ± 0.06
DTFD^35^	82.6 ± 0.56	94.5 ± 0.31	61.0 ± 4.13	56.5 ± 4.67	56.6 ± 5.77	**0.78** ± **0.01**
AMD-MIL^33^	86.0 ± 1.12	94.8 ± 0.13	73.6 ± 3.45	**68.5** ± **1.14**	**66.8** ± **2.55**	0.78 ± 0.03
WiKG^36^	83.1 ± 3.56	95.0 ± 0.42	73.3 ± 1.91	64.6 ± 3.19	62.6 ± 1.50	0.68 ± 0.03
FR-MIL^37^	85.0 ± 0.97	96.0 ± 0.46	**78.3** ± **4.48**	68.0 ± 1.51	65.4 ± 1.22	0.70 ± 0.09
Gigapath^5^ (WSIs pre-trained)						
Max-MIL	83.7 ± 2.77	83.4 ± 3.83	47.3 ± 0.92	45.9 ± 0.76	44.9 ± 1.69	0.75 ± 0.03
Mean-MIL	76.1 ± 4.24	81.3 ± 0.55	49.7 ± 3.29	49.5 ± 0.91	52.1 ± 1.32	0.51 ± 0.06
ABMIL^19^	81.4 ± 0.49	91.8 ± 0.70	66.6 ± 2.55	61.6 ± 2.21	59.8 ± 2.55	0.75 ± 0.03
Gate-ABMIL^19^	81.4 ± 1.73	92.7 ± 0.73	70.6 ± 1.69	63.2 ± 2.80	61.1 ± 2.50	0.72 ± 0.07
CLAM-SB^25^	78.7 ± 2.33	92.6 ± 0.38	59.9 ± 2.54	54.8 ± 1.68	55.2 ± 4.44	0.72 ± 0.08
CLAM-MB^25^	84.4 ± 1.92	**96.5** ± **0.44**	**81.1** ± **3.75**	**68.5** ± **0.39**	65.6 ± 0.30	0.64 ± 0.09
DSMIL^34^	86.2 ± 0.85	93.5 ± 0.61	68.8 ± 4.85	65.2 ± 3.73	63.8 ± 3.42	0.75 ± 0.06
TransMIL^24^	**86.2** ± **0.76**	95.6 ± 0.36	73.4 ± 4.51	67.9 ± 4.69	**66.5** ± **4.56**	**0.80** ± **0.02**
DTFD^35^	80.9 ± 1.18	93.4 ± 0.81	58.1 ± 2.52	52.7 ± 1.53	53.5 ± 1.32	0.76 ± 0.02
AMD-MIL^33^	84.6 ± 1.20	95.0 ± 0.41	72.9 ± 1.31	66.4 ± 1.12	64.9 ± 2.85	0.75 ± 0.07
WiKG^36^	83.8 ± 1.41	94.4 ± 0.52	73.8 ± 2.83	66.0 ± 2.31	63.8 ± 1.39	0.74 ± 0.04
FR-MIL^37^	85.9 ± 0.88	95.4 ± 0.89	79.7 ± 6.78	69.2 ± 2.62	65.7 ± 1.82	0.72 ± 0.04

### Camelyon^+^ Benchmark Experiment

**Fig. 5:**
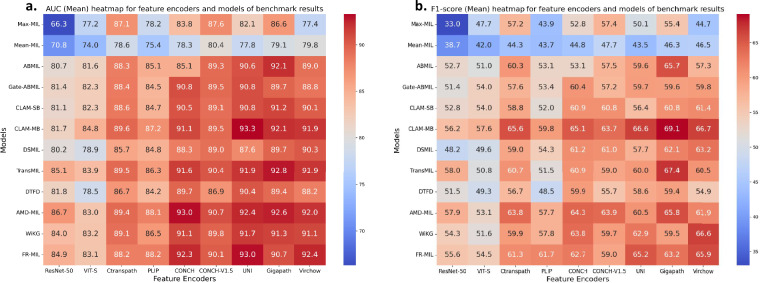
Benchmark comparison of MIL models with various feature encoders. **(a)** Mean AUC scores across 12 MIL models and 9 feature encoders, including ResNet-50^26^, ViT-S^12^, Ctranspath^6^, PLIP^3^, CONCH^7^, CONCH-V1.5^21^, UNI^4^, Gigapath^5^, and Virchow^14^. **(b)** Mean F1-score under the same evaluation setup. Each cell represents the averaged performance across datasets. Warmer colors denote higher values. CLAM-MB^25^, TransMIL^24^, and AMD-MIL^33^ exhibit consistently strong performance across multiple encoders, while newer foundation models such as UNI, Gigapath, and Virchow lead to higher AUC and F1-scores than conventional encoders.

In the Benchmark Experiment on Camelyon^+^^27^, we maintained the same hyperparameter settings as in the comparative experiments on Camelyon-17^23^. On the merged Camelyon^+^ dataset, we evaluated the MIL approach using feature extractors from two pre-trained natural image models, ResNet-50^26^ and VIT-S^12^, and twelve pre-trained pathology image models, Ctranspath, PLIP^3^, CONCH^7^, CONCH-V1.5^21^, UNI^4^, Gigapath^5^, Virchow^14^, Chief^18^, Prism^20^ and Titan^21^. We classify these feature extractors into four main categories: ResNet-50 and ViT-S fall under the domain of natural image pre-training; PLIP, CONCH and CONCH-V1.5 fall under the domain of image-text contrastive learning pre-training; Ctranspath, UNI, GigaPath and Virchow belong to the category of pathology-specific visual pre-training; Chief, Prsim, and Titan are slide-level encoders that can represent a slide as an embedding. We report the mean and standard deviation of model performance in Tables 3–12 which can serve as baselines and references for future work based on the Camelyon^+^ dataset. As shown in Fig. 5, we present a heatmap of the distribution of AUC and F1-score across different MIL models under various feature extractors. It can be observed that pathology-pretrained feature extractors significantly enhance the performance of MIL. Notably, the CONCH model, which uses a VIT-Base architecture with image-text contrastive learning, achieves performance comparable to the UNI and Gigapath models, which utilize VIT-Large and VIT-Giant architectures, respectively. Moreover, both UNI and Gigapath leverage larger training datasets. This suggests that image-text contrastive pretraining may hold greater potential than pure visual pretraining in the pathology domain. While the PLIP model is also pretrained using image-text contrastive learning, its performance does not match that of CONCH, likely due to its smaller dataset and the lower quality of data sourced from Twitter. Slide-level encoders have recently emerged as a popular direction in computational pathology pretraining, aiming to produce holistic representations for entire WSI. However, due to the extremely large resolution of WSIs, the current generation of slide-level encoders still exhibits limited representational capacity. As shown in Table 12, we report the linear probing performance of three representative slide encoders-Chief, Prism, and Titan-and compare them against patch-level encoders (namely Ctranspath, Virchow, and CONCH-V1.5) used for precomputing features in three state-of-the-art MIL frameworks. Among the slide-level encoders, Chief achieved the highest performance, yet still fell significantly short compared to MIL models trained on patch-level features extracted by Ctranspath. This highlights the current limitations of slide-level encoders in capturing the complex pathological patterns required for downstream clinical tasks.

**Table 3:** Results on the Camelyon^+^ dataset with ResNet-50-extracted features.

Methods	Acc (%)	AUC (%)	F1 (%)	Recall (%)	Precision (%)	Kappa
ResNet-50^26^ (ImageNet pre-trained^38^)						
Max-MIL	70.3 ± 7.53	66.3 ± 11.07	33.0 ± 10.65	28.9 ± 12.06	30.8 ± 13.43	0.22 ± 0.30
Mean-MIL	73.2 ± 2.87	70.8 ± 4.14	38.7 ± 3.35	37.9 ± 2.67	41.1 ± 1.77	0.39 ± 0.08
ABMIL^19^	81.1 ± 1.30	80.7 ± 3.32	52.7 ± 1.98	51.5 ± 1.86	52.8 ± 1.98	0.63 ± 0.03
Gate-ABMIL^19^	80.5 ± 1.26	81.4 ± 3.05	51.4 ± 2.05	49.2 ± 3.41	51.2 ± 2.99	0.63 ± 0.03
CLAM-SB^25^	80.2 ± 1.78	81.1 ± 2.93	52.8 ± 2.07	50.9 ± 2.03	51.3 ± 2.51	0.63 ± 0.02
CLAM-MB^25^	**83.5** ± **1.22**	81.7 ± 1.89	56.2 ± 3.80	56.1 ± 2.83	**57.9** ± **1.75**	**0.65** ± **0.03**
DSMIL^34^	79.8 ± 1.05	80.2 ± 4.10	48.2 ± 0.70	46.9 ± 1.67	49.1 ± 4.71	0.61 ± 0.01
TransMIL^24^	81.2 ± 4.49	85.1 ± 1.83	58.0 ± 3.27	**56.5** ± **4.00**	56.8 ± 5.46	0.63 ± 0.03
DTFD^35^	80.6 ± 1.94	81.8 ± 4.04	51.5 ± 2.22	49.7 ± 2.57	51.2 ± 3.36	0.63 ± 0.03
AMD-MIL^33^	81.1 ± 1.91	**86.7** ± **3.43**	**57.9** ± **3.03**	55.8 ± 1.81	55.8 ± 1.81	0.63 ± 0.03
WiKG^36^	81.3 ± 3.30	84.0 ± 3.77	54.3 ± 3.24	54.3 ± 3.60	56.2 ± 3.22	0.62 ± 0.03
FR-MIL^37^	82.8 ± 0.87	84.9 ± 2.97	55.6 ± 2.56	56.4 ± 1.29	58.5 ± 1.63	0.63 ± 0.04

**Table 4:** Results on the Camelyon^+^ dataset with VIT-S-extracted features.

Methods	Acc (%)	AUC (%)	F1 (%)	Recall (%)	Precision (%)	Kappa
VIT-S^12^ (ImageNet pre-trained^38^)						
Max-MIL	76.7 ± 5.30	77.2 ± 1.90	47.7 ± 1.17	45.4 ± 1.97	45.5 ± 6.72	0.58 ± 0.10
Mean-MIL	72.5 ± 4.64	74.0 ± 4.41	42.0 ± 3.99	41.8 ± 4.31	45.4 ± 7.88	0.42 ± 0.07
ABMIL^19^	81.4 ± 2.07	81.6 ± 3.62	51.0 ± 3.26	49.7 ± 4.06	52.9 ± 4.71	0.65 ± 0.04
Gate-ABMIL^19^	78.9 ± 2.05	82.3 ± 3.41	54.0 ± 3.10	53.0 ± 2.78	52.7 ± 2.55	0.61 ± 0.05
CLAM-SB^25^	**81.9** ± **1.52**	82.3 ± 3.57	54.0 ± 3.25	53.2 ± 3.30	57.0 ± 5.80	0.64 ± 0.06
CLAM-MB^25^	81.7 ± 3.08	**84.8** ± **4.11**	**57.6** ± **2.34**	**56.9** ± **2.07**	**59.0** ± **3.74**	0.61 ± 0.09
DSMIL^34^	77.1 ± 3.38	78.9 ± 3.28	49.6 ± 2.83	49.0 ± 2.75	51.2 ± 1.73	0.57 ± 0.07
TransMIL^24^	79.7 ± 1.78	83.9 ± 2.10	50.8 ± 4.61	47.1 ± 5.16	46.7 ± 7.02	0.63 ± 0.03
DTFD^35^	80.4 ± 1.10	78.5 ± 2.96	49.3 ± 2.60	46.4 ± 3.72	47.4 ± 7.18	0.63 ± 0.03
AMD-MIL^33^	81.7 ± 0.72	83.0 ± 2.83	53.1 ± 3.69	51.6 ± 3.35	55.1 ± 3.60	**0.65** ± **0.04**
WiKG^36^	80.8 ± 1.34	83.2 ± 2.79	51.6 ± 2.55	50.1 ± 5.04	51.0 ± 8.07	0.62 ± 0.03
FR-MIL^37^	79.2 ± 3.24	83.1 ± 2.03	54.5 ± 3.56	54.5 ± 1.93	57.4 ± 4.86	0.57 ± 0.06

**Table 5:** Results on the Camelyon^+^ dataset with PLIP-extracted features.

Methods	Acc (%)	AUC (%)	F1 (%)	Recall (%)	Precision (%)	Kappa
PLIP^3^ (WSIs pre-trained)						
Max-MIL	79.4 ± 2.49	78.2 ± 3.44	43.9 ± 1.87	47.3 ± 2.13	41.2 ± 2.01	0.61 ± 0.03
Mean-MIL	73.4 ± 6.31	75.4 ± 5.08	43.7 ± 2.48	43.6 ± 3.44	48.9 ± 3.82	0.50 ± 0.05
ABMIL^19^	81.8 ± 1.94	85.1 ± 4.15	53.1 ± 5.62	54.3 ± 4.08	55.0 ± 5.96	0.66 ± 0.03
Gate-ABMIL^19^	82.4 ± 0.98	84.5 ± 3.91	53.4 ± 1.57	54.7 ± 2.14	54.9 ± 1.68	0.67 ± 0.04
CLAM-SB^25^	81.9 ± 1.81	84.7 ± 4.43	52.0 ± 6.23	53.8 ± 4.15	51.8 ± 8.51	0.65 ± 0.04
CLAM-MB^25^	83.9 ± 0.78	87.2 ± 2.20	59.8 ± 3.83	60.4 ± 4.02	60.9 ± 3.33	0.67 ± 0.04
DSMIL^34^	81.9 ± 2.78	84.8 ± 3.58	54.3 ± 3.03	54.2 ± 2.74	56.1 ± 4.73	0.65 ± 0.02
TransMIL^24^	81.9 ± 1.57	86.3 ± 3.01	51.5 ± 7.23	54.1 ± 5.52	55.4 ± 10.71	0.65 ± 0.04
DTFD^35^	81.3 ± 2.22	84.2 ± 4.23	48.5 ± 4.74	51.4 ± 3.52	49.6 ± 6.55	0.66 ± 0.03
AMD-MIL^33^	**84.9** ± **1.45**	88.1 ± 2.90	57.7 ± 5.05	59.1 ± 4.34	58.9 ± 1.83	**0.69** ± **0.03**
WiKG^36^	82.3 ± 2.34	86.5 ± 4.16	57.8 ± 3.53	58.1 ± 3.31	59.4 ± 3.46	0.61 ± 0.08
FR-MIL^37^	84.6 ± 0.99	**88.2** ± **3.33**	**61.7** ± **5.41**	**62.1** ± **5.62**	**63.9** ± **7.14**	0.67 ± 0.05

**Table 6:** Results on the Camelyon^+^ dataset with CONCH-extracted features.

Methods	Acc (%)	AUC (%)	F1 (%)	Recall (%)	Precision (%)	Kappa
CONCH^7^ (WSIs pre-trained)						
Max-MIL	83.8 ± 3.65	83.8 ± 5.93	52.8 ± 8.29	54.0 ± 6.86	52.8 ± 10.19	0.66 ± 0.06
Mean-MIL	77.9 ± 2.86	78.3 ± 4.54	44.8 ± 2.23	45.7 ± 2.65	46.3 ± 3.95	0.56 ± 0.04
ABMIL^19^	81.8 ± 1.94	85.1 ± 4.15	53.1 ± 5.62	54.3 ± 4.08	55.0 ± 5.96	0.66 ± 0.03
Gate-ABMIL^19^	85.7 ± 1.62	90.8 ± 4.57	60.4 ± 3.07	61.4 ± 3.02	63.0 ± 3.83	0.70 ± 0.06
CLAM-SB^25^	86.6 ± 2.03	90.5 ± 4.97	60.9 ± 1.23	61.8 ± 2.12	64.6 ± 8.59	0.72 ± 0.04
CLAM-MB^25^	**88.0** ± **1.95**	91.1 ± 5.02	**65.1** ± **2.62**	66.6 ± 2.78	66.2 ± 6.34	0.73 ± 0.06
DSMIL^34^	86.8 ± 1.98	88.3 ± 4.21	61.2 ± 1.66	61.5 ± 1.02	61.8 ± 3.03	0.70 ± 0.02
TransMIL^24^	84.7 ± 3.80	91.6 ± 2.73	60.9 ± 3.13	62.6 ± 3.43	64.8 ± 13.42	0.71 ± 0.04
DTFD^35^	86.3 ± 2.68	89.7 ± 4.73	59.9 ± 2.14	61.0 ± 2.00	59.7 ± 3.39	0.72 ± 0.05
AMD-MIL^33^	84.1 ± 3.30	**93.0** ± **3.21**	64.3 ± 6.73	**66.9** ± **8.02**	66.0 ± 5.91	0.62 ± 0.17
WiKG^36^	86.2 ± 2.71	91.1 ± 4.71	63.8 ± 4.29	65.0 ± 4.28	**67.5** ± **12.20**	**0.73** ± **0.03**
FR-MIL^37^	87.3 ± 2.62	92.3 ± 3.16	62.7 ± 3.90	63.7 ± 5.00	63.1 ± 2.65	0.70 ± 0.07

**Table 7:** Results on the Camelyon^+^ dataset with CONCH-V1.5-extracted features.

Methods	Acc (%)	AUC (%)	F1 (%)	Recall (%)	Precision (%)	Kappa
CONCH-V1.5^7^ (WSIs pre-trained)						
Max-MIL	84.7 ± 2.50	87.6 ± 3.29	57.4 ± 2.97	57.2 ± 3.24	59.1 ± 4.59	0.67 ± 0.05
Mean-MIL	79.6 ± 2.11	80.4 ± 3.53	47.7 ± 2.65	47.4 ± 2.81	50.6 ± 5.34	0.62 ± 0.03
ABMIL^19^	84.3 ± 2.43	89.3 ± 2.28	57.5 ± 4.38	57.1 ± 6.17	63.7 ± 6.84	0.68 ± 0.03
Gate-ABMIL^19^	84.4 ± 2.12	89.5 ± 2.70	57.2 ± 2.55	56.1 ± 3.34	58.1 ± 4.10	0.69 ± 0.01
CLAM-SB^25^	86.2 ± 1.95	89.1 ± 3.57	60.8 ± 2.57	60.8 ± 3.82	63.6 ± 7.13	**0.70** ± **0.04**
CLAM-MB^25^	85.7 ± 3.15	89.5 ± 2.99	63.7 ± 4.21	**63.1** ± **3.29**	63.4 ± 2.38	0.65 ± 0.11
DSMIL^34^	84.0 ± 3.29	89.0 ± 2.72	61.0 ± 2.05	60.3 ± 2.32	62.1 ± 5.04	0.66 ± 0.12
TransMIL^24^	80.9 ± 4.45	90.4 ± 1.89	59.0 ± 5.94	56.4 ± 5.58	61.4 ± 4.61	0.61 ± 0.15
DTFD^35^	82.3 ± 2.18	86.9 ± 2.96	55.7 ± 4.51	53.2 ± 5.49	54.1 ± 5.53	0.66 ± 0.06
AMD-MIL^33^	**85.8** ± **2.93**	**90.7** ± **2.55**	**63.9** ± **6.76**	63.0 ± 7.81	**65.5** ± **7.06**	0.68 ± 0.07
WiKG^36^	84.8 ± 2.84	89.8 ± 1.89	59.7 ± 4.94	59.0 ± 6.45	61.2 ± 6.67	0.66 ± 0.09
FR-MIL^37^	84.9 ± 1.15	90.1 ± 1.50	59.0 ± 2.02	59.2 ± 2.69	61.1 ± 3.43	0.69 ± 0.03

**Table 8:** Results on the Camelyon^+^ dataset with Ctranspath-extracted features.

Methods	Acc (%)	AUC (%)	F1 (%)	Recall (%)	Precision (%)	Kappa
Ctranspath^6^ (WSIs pre-trained)						
Max-MIL	83.9 ± 2.34	87.1 ± 4.75	57.2 ± 3.55	57.2 ± 3.12	58.6 ± 2.81	0.63 ± 0.06
Mean-MIL	75.8 ± 2.56	78.6 ± 4.28	44.3 ± 4.14	43.9 ± 4.47	46.3 ± 4.96	0.52 ± 0.05
ABMIL^19^	84.3 ± 1.09	88.3 ± 4.47	60.3 ± 2.48	60.4 ± 2.05	65.7 ± 9.03	0.68 ± 0.03
Gate-ABMIL^19^	84.1 ± 1.99	88.4 ± 4.26	57.6 ± 3.69	56.4 ± 3.20	58.0 ± 3.14	0.68 ± 0.04
CLAM-SB^25^	84.4 ± 2.14	88.6 ± 3.40	58.8 ± 2.51	57.8 ± 2.20	57.7 ± 2.85	0.68 ± 0.03
CLAM-MB^25^	**87.4** ± **1.38**	**89.6** ± **3.84**	**65.6** ± **5.08**	**65.2** ± **5.53**	**66.1** ± **7.02**	**0.72** ± **0.03**
DSMIL^34^	84.8 ± 2.18	85.7 ± 4.83	59.0 ± 3.49	59.1 ± 2.81	60.1 ± 2.19	0.65 ± 0.06
TransMIL^24^	85.3 ± 3.28	89.5 ± 3.42	60.7 ± 9.28	58.9 ± 12.26	60.8 ± 15.92	0.71 ± 0.05
DTFD^35^	83.9 ± 2.68	86.7 ± 5.76	56.7 ± 6.00	55.3 ± 6.22	56.5 ± 4.28	0.68 ± 0.04
AMD-MIL^33^	85.9 ± 2.30	89.4 ± 3.69	63.8 ± 4.96	63.7 ± 5.09	64.8 ± 6.67	0.68 ± 0.08
WiKG^36^	85.6 ± 2.21	89.1 ± 2.22	59.9 ± 3.34	59.6 ± 3.21	60.3 ± 2.93	0.67 ± 0.06
FR-MIL^37^	84.7 ± 2.45	88.2 ± 3.86	61.3 ± 1.81	61.4 ± 2.35	62.0 ± 1.69	0.63 ± 0.06

**Table 9:** Results on the Camelyon^+^ dataset with UNI-extracted features.

Methods	Acc (%)	AUC (%)	F1 (%)	Recall (%)	Precision (%)	Kappa
UNI^4^ (WSIs pre-trained)						
Max-MIL	81.1 ± 3.77	82.1 ± 5.87	50.1 ± 7.48	52.1 ± 7.05	50.1 ± 7.47	0.63 ± 0.06
Mean-MIL	74.1 ± 4.15	77.8 ± 3.53	43.5 ± 4.51	44.4 ± 3.81	43.8 ± 5.30	0.49 ± 0.08
ABMIL^19^	84.6 ± 2.28	90.6 ± 2.94	59.6 ± 2.12	60.2 ± 1.62	60.4 ± 2.97	0.69 ± 0.06
Gate-ABMIL^19^	84.1 ± 3.13	90.8 ± 1.01	59.7 ± 4.00	60.3 ± 3.41	63.0 ± 5.87	0.70 ± 0.04
CLAM-SB^25^	83.5 ± 3.41	90.8 ± 3.82	56.4 ± 3.59	57.5 ± 2.91	56.8 ± 4.38	0.68 ± 0.03
CLAM-MB^25^	**88.0** ± **1.93**	**93.3** ± **3.40**	**66.6** ± **5.70**	**67.1** ± **6.87**	70.2 ± 5.74	**0.75** ± **0.04**
DSMIL^34^	83.0 ± 3.76	87.6 ± 3.01	57.7 ± 5.76	56.7 ± 5.76	64.7 ± 13.70	0.65 ± 0.05
TransMIL^24^	85.9 ± 2.32	91.9 ± 2.59	60.0 ± 10.56	62.0 ± 9.04	66.8 ± 13.03	0.72 ± 0.03
DTFD^35^	84.4 ± 2.97	90.4 ± 2.93	58.6 ± 4.08	59.6 ± 2.76	60.0 ± 7.16	0.72 ± 0.05
AMD-MIL^33^	85.3 ± 1.76	92.4 ± 1.19	60.5 ± 6.29	61.4 ± 6.37	61.7 ± 6.59	0.72 ± 0.03
WiKG^36^	85.6 ± 3.36	91.7 ± 2.31	62.9 ± 3.13	64.3 ± 2.39	64.2 ± 8.35	0.73 ± 0.03
FR-MIL^37^	85.2 ± 2.81	93.0 ± 2.20	65.2 ± 4.46	64.9 ± 5.57	**70.9** ± **9.53**	0.69 ± 0.05

**Table 10:** Results on the Camelyon^+^ dataset with Gigapath-extracted features.

Methods	Acc (%)	AUC (%)	F1 (%)	Recall (%)	Precision (%)	Kappa
Gigapath^5^ (WSIs pre-trained)						
Max-MIL	81.4 ± 6.62	86.6 ± 6.96	55.4 ± 5.93	54.6 ± 5.35	54.9 ± 5.53	0.65 ± 0.11
Mean-MIL	77.4 ± 3.20	79.1 ± 3.17	46.3 ± 4.24	46.3 ± 4.61	53.6 ± 9.24	0.53 ± 0.09
ABMIL^19^	79.2 ± 2.43	92.1 ± 0.27	65.7 ± 6.11	58.1 ± 5.31	58.6 ± 0.99	0.68 ± 0.03
Gate-ABMIL^19^	84.9 ± 3.00	89.7 ± 3.41	59.6 ± 1.61	58.7 ± 2.70	59.5 ± 4.52	0.69 ± 0.04
CLAM-SB^25^	85.4 ± 2.93	91.2 ± 2.47	60.8 ± 3.78	59.8 ± 3.56	63.7 ± 8.90	0.72 ± 0.03
CLAM-MB^25^	86.7 ± 3.01	92.1 ± 2.88	**69.1** ± **8.13**	66.7 ± 5.36	67.0 ± 3.40	0.70 ± 0.07
DSMIL^34^	86.5 ± 3.12	89.7 ± 4.70	62.1 ± 4.48	62.3 ± 2.86	64.2 ± 2.46	0.68 ± 0.06
TransMIL^24^	**88.2** ± **2.77**	**92.8** ± **2.44**	67.4 ± 3.54	**66.9** ± **2.68**	75.5 ± 11.95	**0.73** ± **0.07**
DTFD^35^	83.7 ± 2.33	89.4 ± 4.64	59.4 ± 3.78	58.0 ± 4.24	61.3 ± 3.57	0.68 ± 0.07
AMD-MIL^33^	87.3 ± 1.92	92.6 ± 3.11	65.8 ± 4.68	64.6 ± 4.28	64.6 ± 4.42	0.72 ± 0.05
WiKG^36^	85.0 ± 3.89	91.3 ± 2.93	59.5 ± 5.13	59.4 ± 6.88	68.0 ± 16.12	0.72 ± 0.06
FR-MIL^37^	86.2 ± 2.01	90.7 ± 3.11	63.2 ± 3.54	63.8 ± 2.40	**75.7** ± **10.20**	0.71 ± 0.06

**Table 11:** Results on the Camelyon^+^ dataset with Virchow-extracted features.

Methods	Acc (%)	AUC (%)	F1 (%)	Recall (%)	Precision (%)	Kappa
Virchow^14^ (WSIs pre-trained)						
Max-MIL	73.8 ± 7.04	77.4 ± 5.15	44.7 ± 2.26	42.4 ± 1.08	40.7 ± 2.55	0.53 ± 0.07
Mean-MIL	72.4 ± 8.91	79.8 ± 4.81	46.5 ± 5.48	46.0 ± 5.80	48.0 ± 8.04	0.53 ± 0.05
ABMIL^19^	84.3 ± 2.75	89.0 ± 4.22	57.3 ± 2.56	55.6 ± 5.26	58.9 ± 2.43	0.67 ± 0.03
Gate-ABMIL^19^	84.7 ± 3.74	88.8 ± 3.63	59.8 ± 3.95	59.7 ± 5.05	63.0 ± 9.50	0.67 ± 0.07
CLAM-SB^25^	85.2 ± 2.88	90.1 ± 4.31	61.4 ± 2.42	60.9 ± 2.49	63.2 ± 4.47	0.67 ± 0.08
CLAM-MB^25^	87.0 ± 1.33	91.9 ± 4.67	**66.7** ± **8.64**	64.5 ± 6.30	65.0 ± 4.65	0.70 ± 0.04
DSMIL^34^	86.3 ± 2.00	90.3 ± 4.18	63.2 ± 4.85	63.6 ± 4.29	65.2 ± 3.85	0.65 ± 0.07
TransMIL^24^	85.9 ± 1.83	91.9 ± 3.20	60.5 ± 7.53	59.3 ± 6.78	63.6 ± 4.99	0.70 ± 0.04
DTFD^35^	81.8 ± 3.52	88.2 ± 4.26	54.9 ± 4.26	52.6 ± 7.02	54.7 ± 10.42	0.61 ± 0.13
AMD-MIL^33^	86.3 ± 3.00	92.0 ± 3.71	61.9 ± 6.22	59.9 ± 4.77	60.2 ± 2.78	0.72 ± 0.07
WiKG^36^	86.3 ± 3.86	91.1 ± 4.33	66.6 ± 4.04	65.2 ± 2.86	64.9 ± 2.75	0.67 ± 0.10
FR-MIL^37^	**87.7** ± **2.18**	**92.4** ± **2.13**	65.9 ± 3.83	**67.3** ± **4.13**	**65.4** ± **3.32**	**0.71** ± **0.05**

**Table 12:** Performance comparison between the slide encoder and its corresponding patch encoder.

Methods	Acc (%)	AUC (%)	F1 (%)	Recall (%)	Precision (%)	Kappa
Ctranspath^6^ and Chief^18^ (WSIs pre-trained)						
CLAM-MB^25^	**87.4** ± **1.38**	**89.6** ± **3.84**	**65.6** ± **5.08**	**65.2** ± **5.53**	**66.1** ± **7.02**	**0.72** ± **0.03**
AMD-MIL^33^	85.9 ± 2.30	89.4 ± 3.69	63.8 ± 4.96	63.7 ± 5.09	64.8 ± 6.67	0.68 ± 0.08
FR-MIL^37^	84.7 ± 2.45	88.2 ± 3.86	61.3 ± 1.81	61.4 ± 2.35	62.0 ± 1.69	0.63 ± 0.06
Chief^37^	81.2 ± 0.47	79.3 ± 4.08	48.4 ± 2.46	49.0 ± 1.32	52.1 ± 5.30	0.62 ± 0.03
Virchow^14^ and Prism^20^ (WSIs pre-trained)						
CLAM-MB^25^	87.0 ± 1.33	91.9 ± 4.67	**66.7** ± **8.64**	64.5 ± 6.30	65.0 ± 4.65	0.70 ± 0.04
AMD-MIL^33^	86.3 ± 3.00	92.0 ± 3.71	61.9 ± 6.22	59.9 ± 4.77	60.2 ± 2.78	**0.72** ± **0.07**
FR-MIL^37^	**87.7** ± **2.18**	**92.4** ± **2.13**	65.9 ± 3.83	**67.3** ± **4.13**	**65.4** ± **3.32**	0.71 ± 0.05
Prism^37^	66.4 ± 9.18	70.2 ± 4.23	43.6 ± 4.57	43.3 ± 4.06	46.9 ± 3.30	0.41 ± 0.11
CONCH-V1.5^21^ and Titan^21^ (WSIs pre-trained)						
CLAM-MB^25^	85.7 ± 3.15	89.5 ± 2.99	63.7 ± 4.21	**63.1** ± **3.29**	63.4 ± 2.38	0.65 ± 0.11
AMD-MIL^33^	**85.8** ± **2.93**	**90.7** ± **2.55**	**63.9** ± **6.76**	63.0 ± 7.81	**65.5** ± **7.06**	0.68 ± 0.07
FR-MIL^37^	84.9 ± 1.15	90.1 ± 1.50	59.0 ± 2.02	59.2 ± 2.69	61.1 ± 3.43	**0.69** ± **0.03**
Titan^37^	73.6 ± 3.85	70.5 ± 4.66	41.1 ± 3.12	40.9 ± 3.62	49.3 ± 9.16	0.45 ± 0.07

**Fig. 6:**
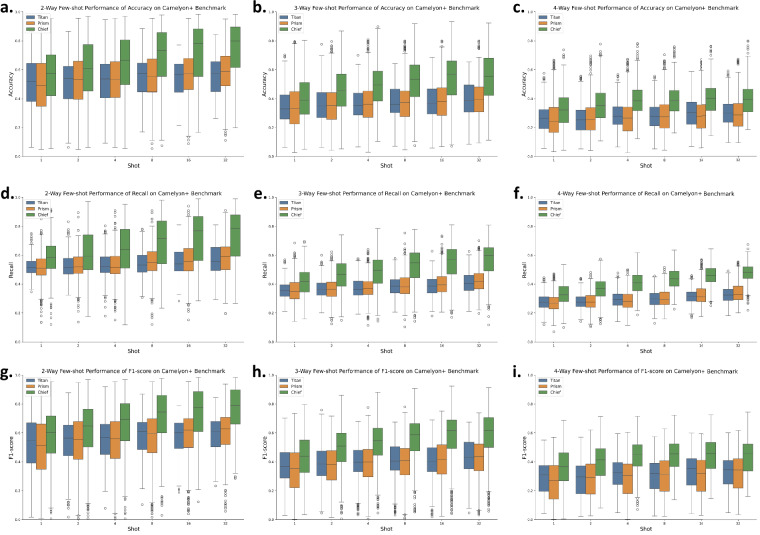
Few-shot classification performance on the Camelyon+^27^. Benchmark. Boxplots show the performance of three models (Titan^21^, Prism^20^, Chief^18^) under 2-way, 3-way, and 4-way classification settings, with varying numbers of shots (1, 2, 4, 8, 16, 32). **(a–c)** Accuracy, **(d–f)** Recall, and **(g–i)** F1-score across increasing few-shot levels. Each box represents model performance distribution over multiple test episodes. Titan and Chief generally outperform Prism in low-shot regimes, and the performance gap narrows with increasing shot numbers.

Figure 6 further presents few-shot learning results using slide encoders under various N-way, K-shot settings, ranging from 1 to 32 shots. The observed performance consistently improves with increasing shot number, suggesting that expanding annotated datasets for tasks such as lymph node metastasis detection could meaningfully enhance the clinical utility of slide-level foundation models.

**Fig. 7:**
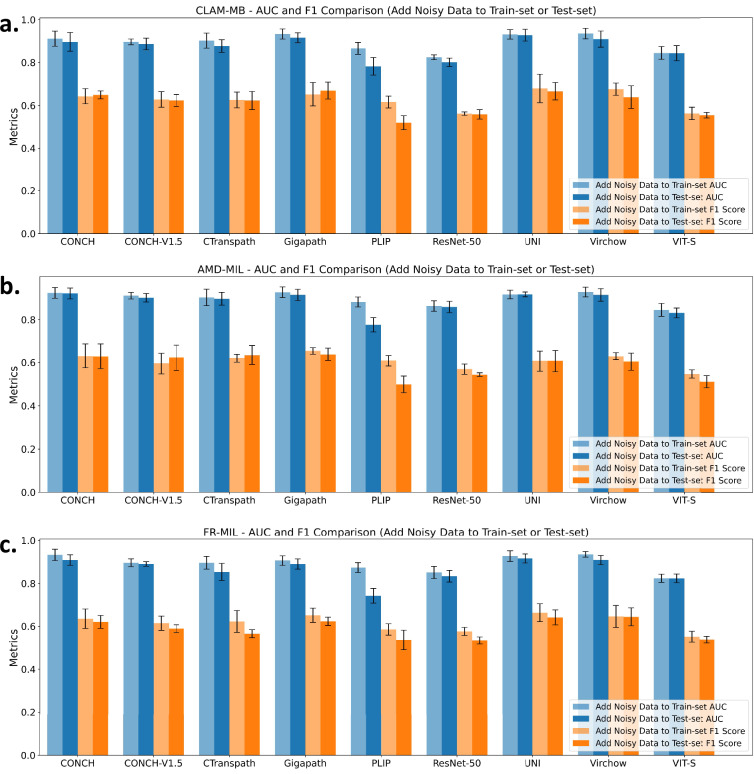
Impact of noisy data on model performance across different feature encoders. Bar plots show the AUC and F1-score of three MIL models **(a)** CLAM-MB^25^, **(b)** AMD-MIL^33^, and **(c)** FR-MIL^37^-when noisy data is added either to the training set or to the test set. Each group of bars compares the performance across multiple feature encoders, including ResNet-50^26^, ViT-S^12^, PLIP^3^, CONCH^7^, CONCH-V1.5^21^, Ctranspath^6^, Gigapath^5^, UNI^4^, and Virchow^14^. Models exhibit more robust performance degradation when noise is added to the test set, while training set noise leads to more varied effects depending on the encoder.

In the Camelyon^+^^27^ dataset, noisy samples were initially removed to construct a clean benchmark. To assess the impact of noisy data, we conducted comparative experiments by reintroducing the noisy subset into either the training set or the test set of Camelyon^+^. As shown in Fig. 7, we evaluated the performance under 9 patch-level encoders and 3 state-of-the-art MIL methods. We observed that, in most cases, adding noisy data to the training set while keeping the test set clean resulted in performance comparable to the original benchmark. In contrast, when the test set was augmented with noisy data while keeping the training set unchanged, the performance on the test set dropped significantly compared to the benchmark. These results suggest that while incorporating noisy data during training may improve robustness without harming performance, the presence of noise in the evaluation set substantially undermines the reliability of performance estimation. This highlights the importance of clean and well-curated test data when benchmarking and deploying pathology AI systems.

**Fig. 8:**
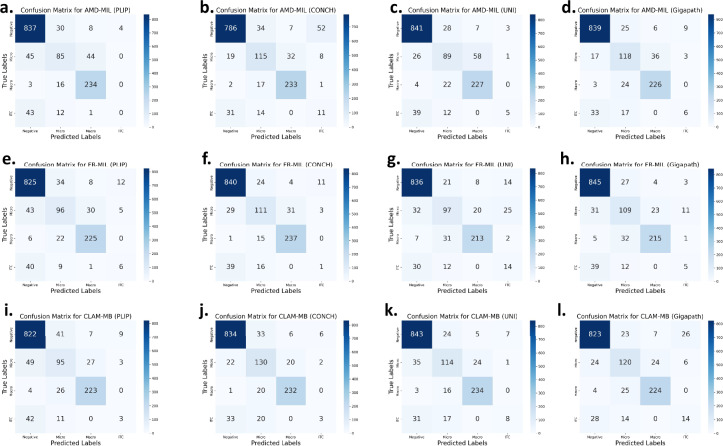
Confusion matrices of MIL models on the four-class metastasis classification task. Each subfigure presents the confusion matrix of a model-encoder pair on the Camelyon^+^^27^ benchmark. Rows indicate the ground truth labels and columns indicate the predicted labels. **(a–d)** show results for AMD-MIL^33^ combined with PLIP^3^, CONCH^7^, UNI^4^, and Gigapath^5^ encoders, respectively. **(e–h)** show results for FR-MIL^37^, and **(i–l)** for CLAM-MB^25^ under the same set of encoders.

In the benchmark results, while the model demonstrates relatively strong performance in terms of accuracy and AUC, the F1-score, recall, and precision are notably low. As illustrated in Fig. 8, we visualized the confusion matrices for the CLAM-MB^25^ and FR-MIL^37^ models. The results show that the models perform relatively well in classifying the negative, micro, and macro categories, but perform poorly in the ITC category. We used macro-averaging to calculate the F1-score, recall, and precision, and the model’s poor performance on the ITC category significantly lowered the overall performance metrics. To investigate this issue further, we analyzed the model’s difficulty in identifying ITC cases. One major factor is the severe class imbalance in the Camelyon^+^^27^ dataset. As shown in Fig. 2, the head class, negative, contains 871 slides, while the tail class, ITC, contains only 54 slides, resulting in an imbalance ratio of approximately 16.1. This imbalance classifies the dataset as having a moderately long-tailed distribution. Such imbalance highlights a key challenge in pathology image analysis: how to achieve balanced model performance on long-tailed datasets like Camelyon^+^, particularly since real-world pathological data naturally follow a long-tailed distribution. Furthermore, we identified a fundamental difference between the four-class classification task in Camelyon^+^ and typical cancer subtyping tasks. The ITC, micro, and macro categories in Camelyon^+^ are primarily distinguished by the size of metastatic regions, whereas MIL is generally better suited for binary classification tasks, such as detecting the presence or absence of cancer. This explains why models achieve high performance on binary tasks like those in Camelyon-16^22^ or Camelyon-17^23^. Consequently, Camelyon^+^ raises an important question about whether the MIL approach the most suitable paradigm for clinical classification tasks like Camelyon^+^, where categories are defined by the size of metastatic regions rather than distinct subtypes of cancer.

### Evaluation metrics

In the comparative experiments on the Camelyon-17^23^ dataset and in the benchmark evaluations, we used accuracy, AUC, F1-score, recall, precision, and Cohen’s kappa coefficient to assess classification performance. The kappa coefficient is a statistical metric used to evaluate the level of agreement between predicted and true labels. It is particularly useful for assessing the performance of classification models in multi-class settings, as it accounts for agreement that may occur by chance.

## Usage Notes

The Camelyon^+^^27^ Dataset is publicly available under the Creative Commons Zero (CC0) license. However, please note that this dataset is not intended for developing diagnosis-focused algorithms or models, and should not be used as the sole basis for clinical evaluations in classification tasks.

## Data Availability

The code related to dataset partitioning strategies, hyperparameter configurations, integration of MIL methods, and evaluation metric calculations is available at: https://github.com/lingxitong/MIL_BASELINE.
